# Conjunctival infiltrates and cytokines in an experimental immune-mediated blepharoconjunctivitis rat model

**DOI:** 10.3389/fmed.2023.1200589

**Published:** 2023-06-28

**Authors:** Aihua Hou, Min Qi Tin, Beau Fenner, Yu-Chi Liu, Louis Tong

**Affiliations:** ^1^Ocular Surface Research Group, Singapore Eye Research Institute, Singapore, Singapore; ^2^Ophthalmology and Visual Sciences, Academic Clinical Programme, Duke-NUS Medical School, Singapore, Singapore; ^3^Department of Medical Retina, Singapore National Eye Centre, Singapore, Singapore; ^4^Corneal and External Eye Disease Service, Singapore National Eye Centre, Singapore, Singapore; ^5^Yong Loo Lin School of Medicine, National University of Singapore, Singapore, Singapore

**Keywords:** allergic blepharoconjunctivitis, Sprague–Dawley, rat model, cytokines, conjunctival infiltrates

## Abstract

**Purpose:**

To characterize the histopathological and immunological findings of a rat model of allergic blepharoconjunctivitis (BC) and demonstrate its potential utility for the assessment of BC therapies.

**Methods:**

Sprague–Dawley (SD) rats were immunized with ovalbumin (OVA) and topically challenged with OVA (BC group) or PBS (control group), while a corticosteroid group was pre-treated with triamcinolone acetate 24 h before the challenge. Morphological features were evaluated and tissues were harvested for histological, flow cytometry and cytokine analysis.

**Results:**

The BC group rats developed eyelid excoriations, redness, and conjunctival edema 24 h after the OVA challenge, while corticosteroid pre-treated and PBS-challenged rats were unaffected. The BC features were reduced despite repeated challenges for 5 days. Massive immune cell infiltration was observed in conjunctivae of BC rats, while no significant infiltration was seen in the other groups. Populations of T cells, mono-macrophages, neutrophils, and NK cells made up more than 77% of CD45^+^7AAD^−^ cells in the conjunctival tissues. T cell proportions were increased at 96 h compared to 24 h post-challenge, while macrophages decreased during the same time period. Eosinophils and intraepithelial neutrophils were detected in the BC rats, but not in the PBS and corticosteroid groups. BC eyes had significantly higher levels of IFN-γ and IL-2, while IL-4 and IL-6 levels were similar to controls.

**Conclusion:**

A robust BC response was detected in this rat model which was suppressed by corticosteroid pre-treatment. Immune cell composition and cytokine profiles changed over time.

## 1. Introduction

Allergic blepharoconjunctivitis (BC) is a common ocular condition characterized by inflammation of the conjunctiva, cornea, and eyelids. Symptoms of allergic BC include tearing, itching, pain or swelling, and in more severe cases, corneal edema, scarring, or secondary infections leading to vision loss (1–3). Experimental models of immune-mediated allergy have been established to assess the immunological mechanisms of the disease and test new therapeutic approaches (4–8). Because of their larger eyeball sizes compared to those of mice, rats are one of the most commonly used laboratory animals for the assessment of ocular allergies.

Genetic differences between rat strains complicate their use as allergic disease models, as their immune responses depend on their genetic background (6, 7). In Lewis rat models of BC, mononuclear cells are the dominant infiltrating cells in the conjunctiva, whereas eosinophils were prominent in Brown Norway (BN) BC rats. Lewis rats appear to be more susceptible to Th1-mediated immune responses, whereas BN rats develop Th2-mediated reactions (6, 7).

Sprague Dawley (SD) rats are a widely used research strain. Although the immune-mediated BC model in SD rats has been used to evaluate anti-inflammatory molecules (9), their ocular immune responses have not been characterized. In humans, there is a need to develop an eye model that focuses on Th1 and Th17 as diseases such as dry eye (which has blepharitis or blepharoconjunctivitis) and other ocular surface conditions have a delayed hypersensitivity reaction and primarily involve such Th subsets (10). In the current work, we investigated the clinical features of an antigen-specific delayed-type hypersensitivity response induced BC model in SD rats. We then profiled the conjunctival cell subpopulations and analyzed the relevant cytokine levels, and assessed the changes in the histological and immune responses over time following the immune challenge. This model can be potentially used for future work on the assessment of BC-related therapies.

## 2. Materials and methods

### 2.1. Immune-mediated blepharoconjunctivitis rat model

The animals were handled according to institutional guidelines and the Association for Research in Vision and Ophthalmology Statement for the Use of Animals in Ophthalmic and Vision Research. The study protocol was approved by the Institutional Animal Care and Use Committee of SingHealth (2016/SHS/1258, 2018/SHS/1448). The rats were housed in the SingHealth Experimental Medicine Centre (Singapore) under standard conditions: room temperature of 21°C–23°C, relative humidity of 45%–50%, and alternating 12-h light–dark cycles (7 a.m. to 7 p.m.). Female Sprague Dawley (SD) rats with ages ranging from 8 to 10 weeks were used in this study.

**Figure 1:**
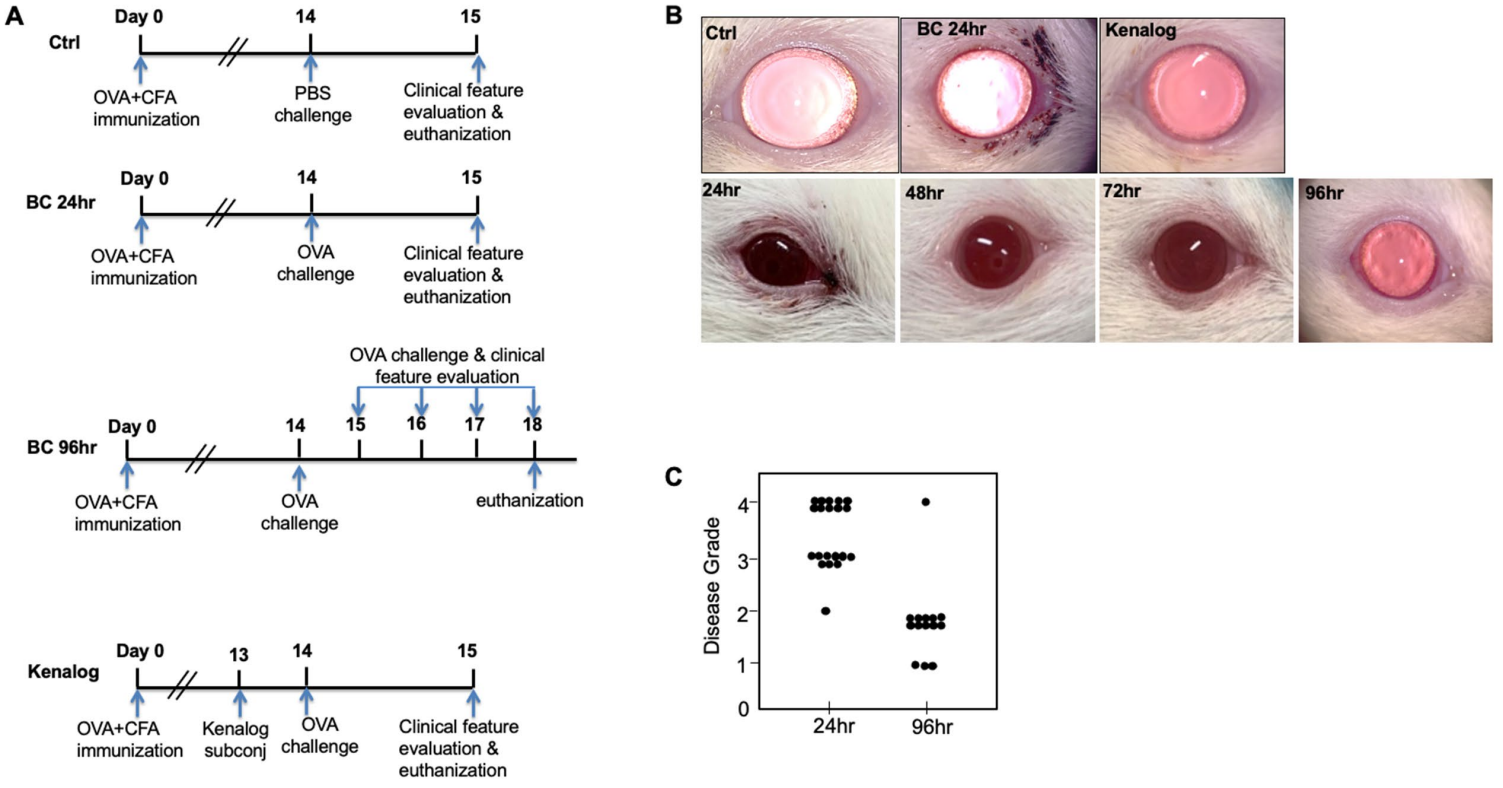
**(A)** Experiment design. Sprague–Dawley rats were subcutaneously immunized with OVA at Day 0. Two weeks later, immunized rats were either topically challenged with OVA or PBS eye drops. Clinical features were evaluated every day thereafter. **(B)** Top row: representative images of rat eyes at 24 h after first topical challenge. Bottom row: representative images of repeatedly challenged rat eyes at different time points. **(C)** BC clinical disease scores at 24 and 96 h. Control, PBS challenged group.

An immune-mediated BC model was replicated in SD rats according to a previously published protocol with minor changes (7, 11). Briefly, a total of 62 rats were injected subcutaneously at the base of the tail with 100 μg of ovalbumin (OVA, Sigma-Aldrich, Singapore) in complete Freund’s adjuvant (CFA, BD Bioscience, Singapore). Two weeks later, the immunized rats were topically challenged in both eyes with either 10 μL of PBS (control group) or 250 μg/10 μL of OVA dissolved in PBS (BC group). Twenty-four hours later, the eyes of the rats were clinically evaluated and imaged with a slit lamp. The rats from the control and 24 h BC groups were then sacrificed. The 96 h BC group was repeatedly challenged for 4 more days, once per day. Clinical features were documented by daily imaging. A corticosteroid group was pre-treated with 400 μg/10 μL of subconjunctival triamcinolone acetate (Kenalog, Bristol-Myers Squibb, New York, United States) given 1 day before the OVA challenge (Figure 1A).

Clinical features were graded according to a previous scoring system (12): both eyes of one rat were assessed for lid swelling and discharge, and each finding was scored as 1 point. For example, if one eye had lid swelling and discharge, the grading score of this eye was 2. After the slit lamp assessment, the animals were euthanized. The eyeballs and eyelids were harvested and tissue sections were prepared. Conjunctival tissues were harvested for either flow cytometry analysis (control group: 4 rats, 24 h BC group: 8 rats, 96 h BC group: 12 rats) or cytokine multiplex analysis (control group: 5 rats, 24 h BC group: 5 rats, 96 h BC group: 11 rats).

### 2.2. Histology, eosinophil, and immunofluorescence analysis

Rat eyes and eyelids were fixed in Davidson’s Formula for 1 h before transferring to 70% ethanol overnight, followed by sequential soaking in 80% ethanol for 1 h, then 95% ethanol and 100% ethanol and xylene for 2 h each before embedding in paraffin, and sectioned with a HistoCore Multicut (Leica Biosystem, Wetzlar, Germany) at 5 μm thickness. The sections were stained with hematoxylin–eosin (H and E) and visualized with a Nikon Eclipse Ti (Nikon, Tokyo, Japan) microscope. We evaluated the density of conjunctival goblet cells in at least three sections of H and E from at least two animals per group, over the bulbar, forniceal, and palpebral conjunctiva.

Eosinophils were enumerated by staining paraffin sections with a modified Llewellyn’s Sirius red method (13). Briefly, after deparaffinization and hydration, sections were stained with hematoxylin Gill 3 for 30 s, rinsed with tap water for 5 min, followed by a rinse in 100% ethanol for 5 min. After that, sections were stained with 0.5% alkaline Sirius red (Sigma-Aldrich, Singapore) solution overnight, rinsed with water, and dehydrated with 100% ethanol before being cleared with xylene again. Coverslips were mounted using Permount (Sigma-Aldrich, Singapore). Stained sections were visualized with a Nikon Eclipse Ti (Nikon, Tokyo, Japan). Three sections were randomly chosen for each eye, and five views were randomly chosen for each section. Four rats were included from each group.

Neutrophil immunofluorescence staining: Paraffin-embedded sections were deparaffinized by sequential soaking in 100%, 95%, 70%, and 50% ethanol, with two washes of 10 min each. Antigen retrieval was performed by soaking the sections in sodium citrate buffer for 25 min at 95°C–100°C. After that, the sections were blocked with 5% bovine serum albumin (BSA, Sigma-Aldrich, Singapore) in phosphate-buffered saline (PBS, Thermal Fisher, Singapore) for 1 h. The Sections were incubated with anti-myeloperoxidase (MPO; ab9535, 1:50, Abcam, Cambridge, United Kingdom) overnight at 4°C and the nuclei were visualized by mounting the cells in DAPI mounting medium (Santa Cruz Biotechnology, Texas, United States). The stained sections were visualized using a Zeiss Imager Z1 microscope (Carl Zeiss Inc., Oberkochen, Germany).

### 2.3. Flow cytometry analysis

Conjunctival tissues were first minced with surgical scissors in 1 mL of digestion medium (PRMI 1640 with 10% FBS, 0.05 mg/mL collagenase, and 0.1 mg/mL DNase I), and then incubated at 37°C for 1 h. After neutralization with fresh medium (PRMI 1640 with 10% FBS), the digested tissues were passed through a 20G needle attached to a 3 mL syringe several times and then filtered through a 70 μm filter. The resulting single cell suspension was centrifuged and re-suspended in staining buffer (0.2% BSA in PBS). Antibodies were added and the cells were incubated on ice for 30 min. After washing once with staining buffer, the stained cells were analyzed with a BD FACSVerse (New Jersey, United States). Data acquisition and analysis were performed using the BD FACS suite software. APC-Cy7-CD45 (clone OX-1), BV421-CD3 (clone 1F4), and FITC-rat granulocyte-his48 were obtained from BD Pharmingen, Singapore. PE-Cy7-CD45RA (OX-33), PE-CD43, and APC-CD161 were obtained from Biolegend Singapore. Cells were stained with 7-AAD to exclude dead cells. The antibody list and gating strategy followed the published method (14).

### 2.4. Conjunctival cytokine analysis

Conjunctival tissues were minced in multiplex assay buffer (Millipore, Massachusetts, United States) with 1× protease inhibitor cocktail (Sigma-Aldrich, Singapore) and sonicated twice for 30 s at 20% power, with 5 s on and 10 s off cycles (Betatek Inc., Toronto). The sonicates were centrifuged at 4°C for 15 min and the supernatant was transferred to a new tube. Protein concentrations were quantified using a bicinchoninic acid kit (Thermo Fisher, Singapore). Cytokines were quantified using a 9-plex cytokine kit from Millipore (Massachusetts, United States) according to the manufacturer’s instructions (15). Duplicate 25 μL aliquots were used for each sample and the results were calibrated to the protein concentration of each sample.

### 2.5. Statistical analysis

Unpaired *T*-tests were performed for continuous variables at two different time points. Significance was determined at the level of *p* < 0.05.

## 3. Results

### 3.1. Topical OVA rechallenge stimulated conjunctival and eyelid inflammation

**Figure 2:**
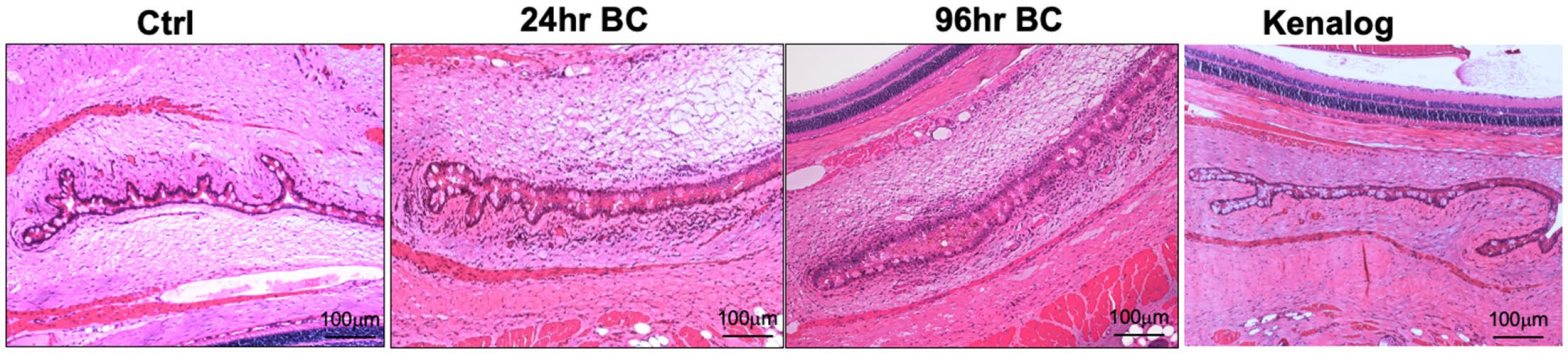
Representative H and E staining images of rat eyes. Massive immune cell infiltration (dark purple, small cells) and conjunctival chemosis with the presence of interstitial fluids in bulbar and palpebral conjunctiva of 24- and 96-h BC rats, while very few immune cells in control and Kenalog eyes. Control, PBS challenged group.

After the topical challenge with OVA, the rats manifested BC with eyelid excoriations and conjunctival edema (Figure 1B). At 24 h post-challenge, eyelid excoriations could be observed in 30 of the 40 challenged eyes, while eyelid and conjunctival edema occurred in all the challenged eyes. Features of BC were not seen in the control eyes challenged with PBS or in those pre-treated with subconjunctival triamcinolone acetate 1 day before the OVA challenge (Figure 1B, upper row). Interestingly, eyelid excoriation became less common over a five-day period despite repeated topical challenges, although eyelid and conjunctival edema was still observed throughout the study duration. Representative images of the same eye at different time points after daily OVA challenges are shown in Figure 1B (lower row). The disease scores of BC rats at 96 h after the initial rechallenge were significantly reduced compared to those at 24 h (1.93 ± 0.73 to 3.45 ± 0.60, *p* < 0.01; Figure 1C). Massive immune infiltrates were observed in the bulbar and palpebral conjunctiva of both the 24- and 96-h BC groups, but there were no obvious immune infiltrates in the conjunctiva of the control and triamcinolone acetate pre-treated groups (Figure 2). A reduction of goblet cells after rechallenging with OVA was observed (Table 1).

**Table 1:** Conjunctival goblet cell density score.

	Bulbar conj^#^	Forniceal conj^#^	Palpebral conj^#^
Control	+	+++	++
24 h BC^*^	+	++	+/++
96 h BC^*^	o/+	+	+
Kenalog	+	+++	++

^#^Conjunctiva; ^*^Blepharoconjunctivitis.

### 3.2. Profiling the subpopulations of immune cells in the conjunctiva of BC rats

**Figure 3:**
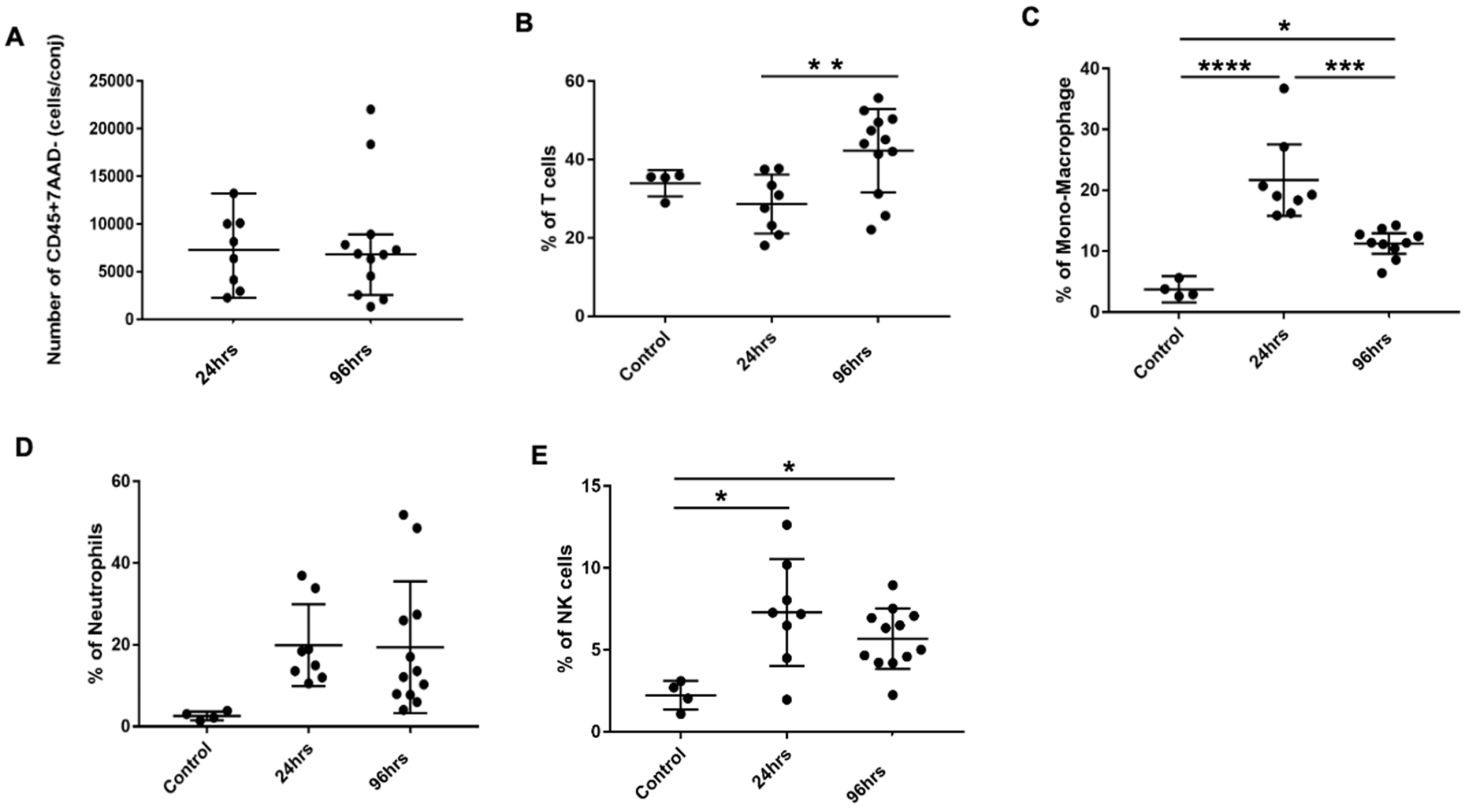
Conjunctival infiltrates in BC rats. **(A)** Total number of CD45^+^7AAD^−^ cells per conjunctiva from 24 and 96h BC rats. **(B–E)** Percentage of conjunctival infiltrates subpopulations by flow cytometry. The Y-axis refers to the percentage of total CD45^+^7AAD^−^ cells. Error bars: SD; *p < 0.05; **p < 0.01; ***p < 0.001; ****p < 0.0001. Control, PBS challenged group.

**Figure 4:**
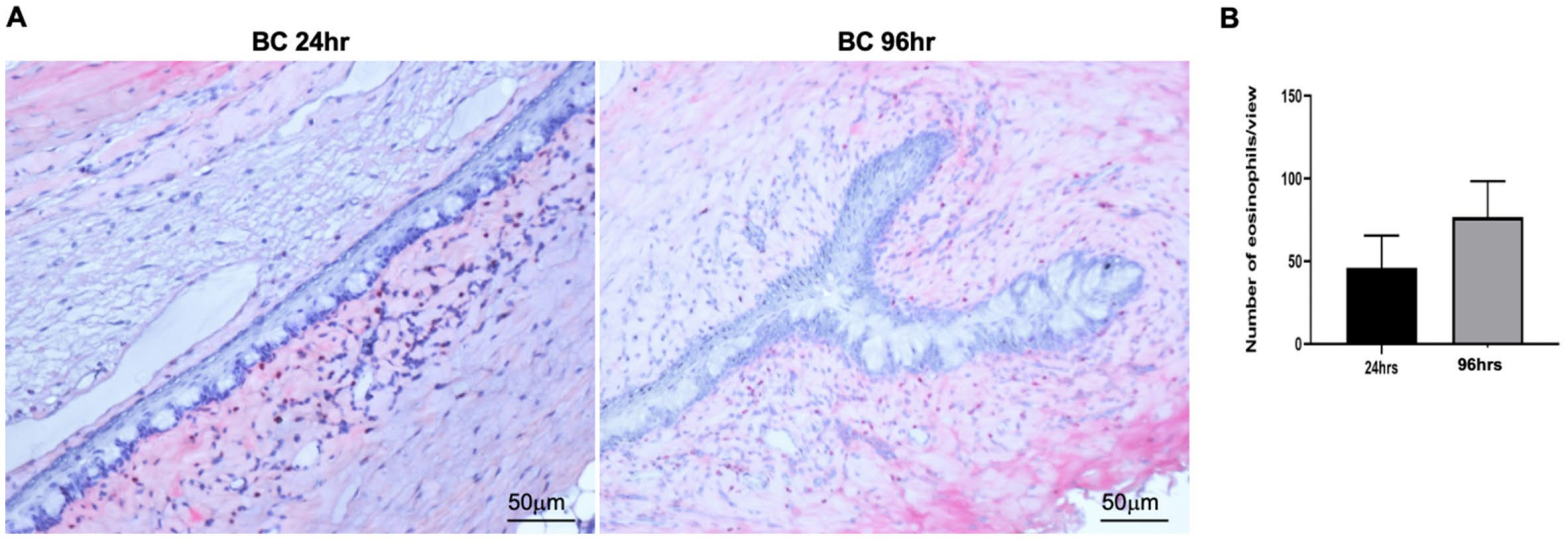
**(A)** Representative images of conjunctival eosinophils in BC rats. Eosinophils were detected by Llewellyn’s Sirius red staining method. Five views were randomly chosen for each section, and three sections were chosen from each rat eye. Red, eosinophils; blue: nuclear. **(B)** Bar chart showing the quantification of eosinophils in the conjunctival sections. Height: mean of 4 rats, Error bars, SD.

We next harvested bulbar conjunctiva from the rats and profiled the subpopulations of immune cells using a published flow cytometry panel (14). The total number of conjunctival CD45^+^7AAD^−^ cells was similar between the 24- and 96-h groups (Figure 3A). T cells, neutrophils, mono-macrophages, and NK cells were collectively the major contributors of CD45^+^7AAD^−^ leukocytes at both time points (Table 2), forming 77.5% ± 5.19% and 77.8% ± 4.78% of total CD45^+^7AAD^−^ leukocytes at the two time points, respectively. On further analysis, the percentage of T cells significantly increased in the 96-h group compared to 24-h group (42.2% ± 10.6% vs. 28.6% ± 7.51%; *p* = 0.005, Figure 3B; Table 2), while the mono-macrophages significantly declined over time (10.5% ± 2.83% at 96 h compared to 21.6% ± 7.02% at 24 h; *p* = 0.0001, Figure 3C; Table 2). The percentages of neutrophils and NK cells were similar at both times (Figures 3D,E; Table 2). The average number of eosinophils in the conjunctival tissues at 24 and 96 h was not statistically different between the two groups (Figures 4A,B).

**Table 2:** Percentage^1^ of various subpopulations of leukocytes within the conjunctiva of rats with blepharoconjunctivitis (BC).

Groups	T cells	Mono-macrophage	Neutrophils	NK cells	Total
24 h (*n* = 8)	28.66 ± 7.51	21.69 ± 7.02	19.89 ± 10.03	7.29 ± 3.26	77.52 ± 5.19
96 h (*n* = 12)	42.29 ± 10.64	10.54 ± 2.83	19.36 ± 16.16	5.70 ± 1.85	77.88 ± 4.78

1 Percentage within the CD45^+^7AAD^−^ population.

### 3.3. Neutrophil transepithelial migration in conjunctivae

**Figure 5:**
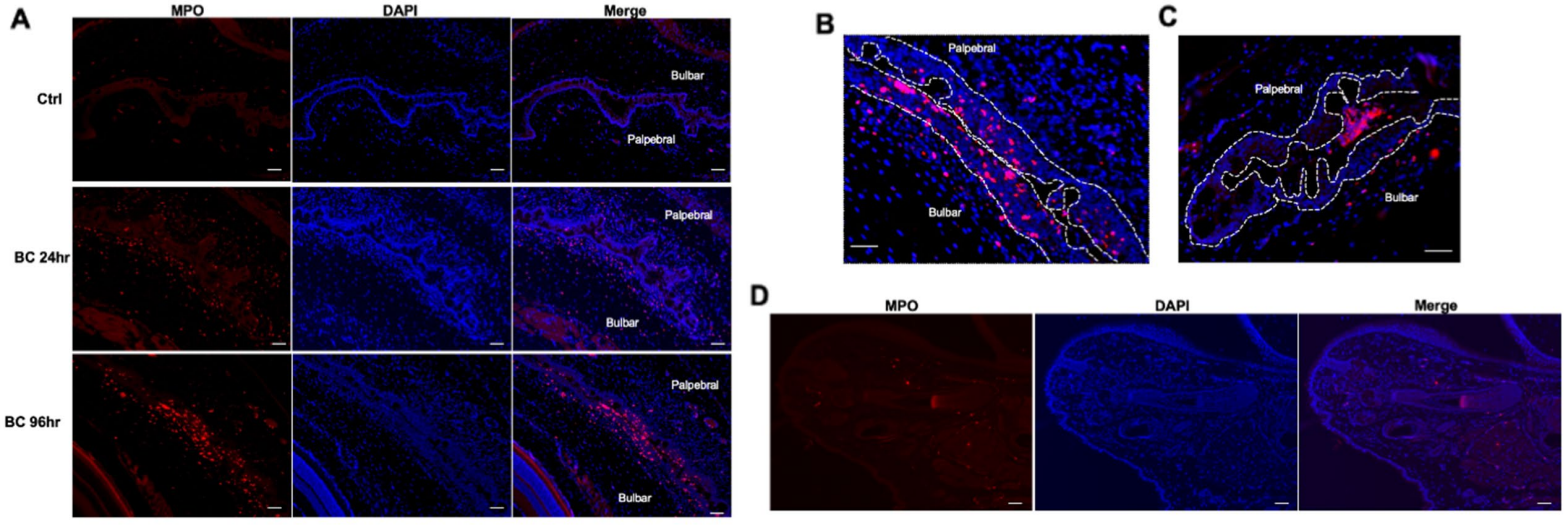
**(A)** Immunofluorescence staining of neutrophils in conjunctival of BC rats. **(B)** Representative images of intraepithelial neutrophils. **(C)** Neutrophil clusters in the tear compartment. **(D)** Microscopic eyelid sections of rats with BC. Red dots: MPO^+^ neutrophils; blue dots: nuclei of cells stained with DAPI. Ctr, PBS challenged group; Scale bar, 50 μm.

Large numbers of MPO-positive neutrophils were observed in the palpebral and bulbar conjunctivae at 24 and 96 h post-challenge, while scanty MPO-positive cells were observed in the conjunctiva of controls (Figure 5A), consistent with flow cytometry results (Figure 3D). Interestingly, significant numbers of neutrophils were located within the palpebral and bulbar conjunctival epithelium, suggesting these cells were migrating across the epithelium (Figure 5B). In some sections, neutrophil clusters were observed within the conjunctival fornix (Figure 5C), suggesting that neutrophils had already migrated through the conjunctival epithelium into the tear film. This phenomenon was observed in the BC rats but not in control rats. In contrast to the bulbar and palpebral conjunctival tissues, very few MPO-positive cells were observed in the eyelids (Figure 5D).

### 3.4. Temporal changes in cytokine profiles

**Figure 6:**
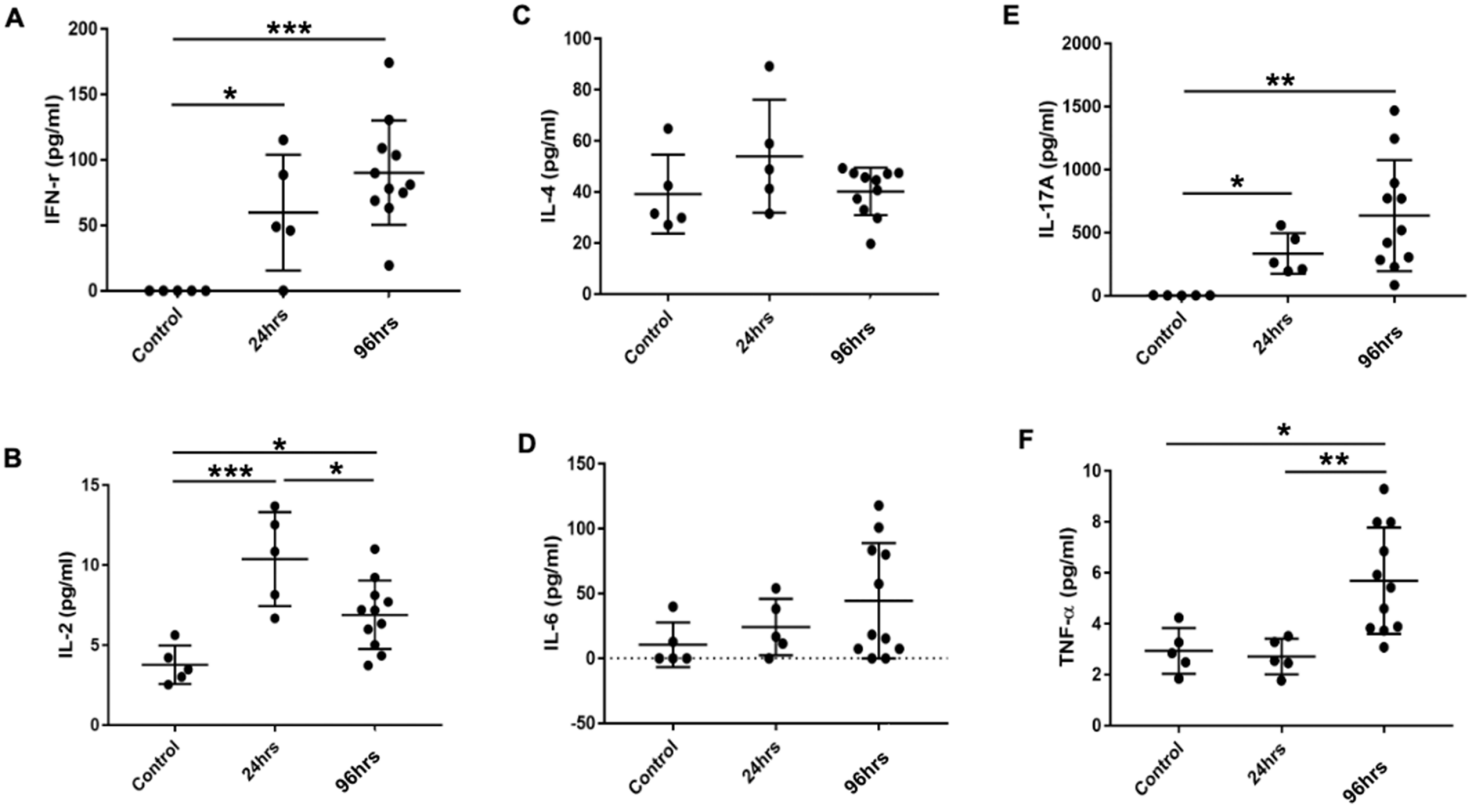
**(A-F)** Cytokines levels in the conjunctiva of BC rats. Conjunctiva tissues harvested and cell suspension was used for cytokine multiplex analysis. Error bars indicate SD, ^*^p < 0.05; ^**^p < 0.01; ^***^p < 0.001. Control: PBS challenged group.

Multiplex cytokine analysis of ocular tissues showed that Th1 cytokines IFN-γ and IL-2 were significantly higher in the OVA-challenged BC rats compared to the controls. IFN-γ levels were higher at 96 h post-challenge compared to 24 h (Figures 6A,B). The levels of Th2 cytokines IL-4 and IL-6 were similar among the three groups of rats (Figures 6C,D), suggesting the immune response in the SD BC rats was mainly Th1-mediated. The Th17 cytokine IL-17 was similarly elevated at 24 and 96 h in the BC rats compared to the controls (Figure 6E) suggesting the presence of a Th17 response. Compared to the controls, TNF-α was not increased in the 24-h BC rats, but was elevated in the 96-h rats (Figure 6F).

## 4. Discussion

A robust clinical and immunologic model of blepharoconjunctivitis was developed in SD rats using systemic OVA sensitization and subsequent topical OVA challenge. The clinical severity of BC was greater at 24 h than at 96 h, despite daily rechallenge topically, although massive immune cell infiltration was observed in bulbar and palpebral conjunctivae at both time points. We did not continue to observe the rats beyond this time for possible cessation of disease signs. T cells, mono-macrophages, neutrophils, NK cells, and eosinophils were profiled in the conjunctival tissues of both 24 and 96 h groups of BC rats. The immune cell profiling showed temporal changes: T cells increased from 24 to 96 h whereas mono-macrophages were decreased. Neutrophil transepithelial migration was observed in conjunctival tissues at both time points. The levels of Th1 cytokines IFN-γ and IL-2 were increased at both time points compared to the controls, while the levels of Th2 cytokines IL-4 and IL-6 were unchanged, suggesting that Th1 reaction was dominant in the SD BC rats, unlike in BN rat models (see Supplementary Datasheet 1). Importantly, pre-treatment with subconjunctival corticosteroid dramatically suppressed the clinical and immunological responses, which demonstrates the utility of this model as an *in vivo* therapeutic screening tool.

The clinical features of BC in this SD rat model were consistent with previous rat BC models (16–18). As in previous reports of BC rat models, the immunized rats were only given one topical OVA rechallenge. Here we found that repeated topical OVA challenges over 5 days did not worsen BC clinical features in the SD rats. The eyelid and conjunctival edema persisted in BC rats after 5 days of rechallenge, but the eyelid excoriations were reduced. This observation could be explained by early-phase and late-phase reactions during ocular allergy (19).

It is possible that the histological features of cellular inflammation do not correlate to morphological signs. When Brown Norway rats were transfused OVA-specific T cells, OVA topical challenge did not induce any clinical signs of disease even though massive infiltration of cells was observed in the conjunctival and eyelid tissues, suggesting that T cell-mediated late-phase reaction evidenced histologically may not be associated with clinical eyelid signs (20, 21). In SD BC rats, topical OVA challenge triggered an early-phase reaction. The itching, burning, and tearing symptoms might have caused scratching behaviors in the rats, explaining the lid excoriations observed at 24 h. After this early phase reaction, the response transited to a later T-cell mediated phase during repeated OVA challenge.

Genetic background determines the immune response features of experimental immune-mediated BC rat models (6, 7). We summarized the immune characteristics of the different animal models reported (Supplementary Datasheet 1). In most studies, infiltrated cells were identified by histological staining and cytokines analyzed by RT-PCR of cultured lymph nodes or by ELISA using lymph nodes cell culture supernatant. In this study, we profiled the infiltrated cell subpopulations and cytokines using conjunctival tissues.

Previous studies showed Th1-mediated allergy in Lewis rats and Th2-mediated immunity in BN rats (6, 7). In the culture supernatant of lymph nodes isolated from BC rats, more IFN-γ was produced in Lewis rats than in BN rats. In contrast, IL-4 was produced only in BN rats and it was below a detectable level in Lewis rats (7). In this study, IFN-γ, IL-2, and IL-17A increased in BC rats, while IL-4 levels showed no difference compared to the controls, indicating the immune responses in SD rats are similar to Lewis rats, i.e., Th1 mediated, but potentially may have an additional Th17 effector arm.

Using histological staining, lymphocytes/mononuclear cells were predominantly found in the conjunctiva of the BC rats with a Lewis background, while eosinophils were predominant in the conjunctiva of the BC rats with a BN background (7, 16). Using a robust 9-color flow-cytometric protocol with minor changes (14), T cells, mono-macrophage, neutrophils, and NK cells were identified in conjunctival tissues of both 24 and 96 h groups of SD rats. Eosinophils were identified by Sirius red staining separately. Our results suggest that T cells dominate the later response, though three immune cell types (T cells, macrophages, and neutrophils) showed similar proportions at 24 h post-challenge (only T cells clearly exceeded 20% at 96 h). Immunohistochemistry staining used in previous studies cannot distinguish macrophage subpopulations (22). In contrast, our flow cytometry protocol identified CD43Lo/His48Hi and CD43Hi/His48Int-Lo monocyte–macrophage populations. Previous studies in rats indicated that CD43Lo rat monocytes are analogous to Ly6CHi murine monocytes, with the capacity to participate in acute inflammatory responses (14, 23, 24). In the LPS pulmonary inflammation rat model, the CD43Lo/His48Hi mono-macrophage population significantly increased in lung tissues 24 h after the challenge (14). In a model of lung allograft rejection, there was a higher number of CD43Lo monocytes isolated directly from the pulmonary vasculature (25). In this SD BC model, only a CD43Lo/His48Hi monocyte-macrophages population was detected, which diminished at 96 h, supporting the concept that CD43Lo/His48Hi monocyte-macrophages participate in acute immune responses.

In an OVA-specific T cell adoptive transfer BC rat model, macrophages were suggested as major antigen-presenting cells during the initial phase of the disease (22). Subconjunctival injection of CL2MDP-lip before the OVA challenge reduced infiltration of macrophages into the conjunctiva during the development of BC. Therefore, CL2MDP-lip has been suggested as a potential therapeutic tool for BC management (18). Preliminary data from a similar experiment in our SD BC rats failed to reduce the clinical features of these rats (Supplementary Figure 1), suggesting that other mechanisms were also involved in the early phase allergy response.

NK cells exert regulatory properties on both innate and adaptive responses and have been reported to increase the conjunctiva of patients with vernal keratoconjunctivitis (26). None of the previous rat BC models studied NK cells in the conjunctiva of BC rats. In OVA-induced mouse models of asthma, depletion of NK cells prevented the generation of OVA-specific IgE, production of type-2 cytokines, and the infiltration of eosinophils and T cells in the lungs, suggesting that NK cells act as agonists of allergic sensitization in allergen-induced models of asthma (27, 28). During the effector phase of allergic response, NK cell depletion increased the number of OVA-specific CD4+ T cells and the levels of IL-23, IL-17A, and IFN-γ (27). In the rat BC model, it is not known if NK cells play a pro- or anti-inflammatory role. There was a slight but not statistically significant drop in proportion from 24 to 96 h. This is an interesting field worth further investigation.

It is well known that neutrophils are involved in BC. In humans, a significant inflammatory cell response consisting mainly of neutrophils was induced in human tears 20 min after an allergen challenge (19). In a di-DNP-L-lysine-induced ocular anaphylaxis SD rat model, neutrophils appeared in rat tear fluid 1 h after a topical challenge (29). Neutrophil transepithelial migration during mucosal inflammation leads to injury and a leaky mucosal barrier (30). Evidence of such migration was also observed in SD BC rats.

We acknowledge several limitations of the current study. First, only T cells, neutrophils, mono-macrophages, and NK cells were profiled using flow cytometry due to antibody and channel limitations. T cell subpopulations, dendritic cells, and other cell populations were not explored. Second, we did not evaluate other allergic reaction-related cytokines at other time points, for example, in one study IL-4 had peak production at 6 and 12 h after a topical challenge, while in an allergic mouse model, IL-4 was observed 30 days after a repeated topical challenge (5, 11). Although Th2 cells are traditionally considered the main mediators of allergic response, other evidence indicated the involvement of Th1 and Th17 cells (31–33). In chronic atopic dermatitis patients, Th1-type immunity has been shown to shift to Th2-type (34). The interaction of T helper subsets in allergic ocular diseases seems to be more complicated, therefore warranting further investigation. Third, we did not observe the course of the disease over the long term after a single rechallenge or rechallenge for a few days. This would be an interesting project but may not be relevant for therapeutic settings. In addition, we did not examine rats at 96 h after a single topical rechallenge and also did not perform PAS staining, but we were able to visualize the goblet cells based on morphology.

In summary, immune-mediated BC can be induced in SD rats, but these have significantly different immune responses to other allergic rat models. Several types of leukocytes were present among the conjunctival infiltrate, with a mainly Th1 and Th17 immune response and an earlier immune response of multiple immune cell types which coincided with maximal clinical disease in the eyelid. The profile of infiltrating cells and cytokines differed at 24 and 96 h. These results may be useful for planning time points and durations when using BC SD rats for the evaluation of anti-inflammatory treatment.

## Data availability statement

The original contributions presented in the study are included in the article/Supplementary material, further inquiries can be directed to the corresponding author.

## Ethics statement

Animals were handled according to institutional guidelines and the Association for Research in Vision and Ophthalmology Statement for the Use of Animals in Ophthalmic and Vision Research. The study protocol was approved by the Institutional Animal Care and Use Committee of SingHealth.

## Author contributions

AH and MT performed the experiments, participant data analysis, and manuscript writing and revision. BF and LT provided study funding and participated in the study design, data interpretation, and manuscript writing and revision. Y-CL participated in manuscript writing and revision. All authors contributed to the article and approved the submitted version.

## Funding

This research was supported by the National Medical Research Council (NMRC) under its NMRC/CSA-SI/0017/2017 and also supported by SERI_Lee Foundation 1584/83/2018 and Eye-ACP grant R1395/81/2016.

## Conflict of interest

The authors declare that the research was conducted in the absence of any commercial or financial relationships that could be construed as a potential conflict of interest.

## Publisher’s note

All claims expressed in this article are solely those of the authors and do not necessarily represent those of their affiliated organizations, or those of the publisher, the editors and the reviewers. Any product that may be evaluated in this article, or claim that may be made by its manufacturer, is not guaranteed or endorsed by the publisher.

## Supplementary material

SUPPLEMENTARY FIGURE 1 **(A,B)** Slit lamp images of rat eyes. Rats were immunized with OVA in CFA. Two weeks later, 24hrs before OVA topical challenge, immunized rats were injected with either PBS or Clodrosome (250µg) at subconjunctiva. Both eyes of these rats were photographed with slit lamp. **(C)** The BC clinical disease score of these rats were graded as the following: each eye was assessed for eyelid excoriations and edema, and each findings was scored as 1 point. Scores from two eyes were added for each rat. **(D)** Representative H and E staining images of rat eyes from PBS and Clodrosome groups.

## References

[1] Fukushima A. Current research progress in allergic conjunctival diseases. Allergol Int. (2020) 69:485–6. doi: 10.1016/j.alit.2020.08.003, PMID: 33008568

[2] Miyazaki D Fukagawa K Okamoto S Fukushima A Uchio E Ebihara N. Epidemiological aspects of allergic conjunctivitis. Allergol Int. (2020) 69:487–95. doi: 10.1016/j.alit.2020.06.004, PMID: 32654975

[3] Bonini S Leonardi A. The multifaceted aspects of ocular allergies: phenotypes and endotypes. Ocul Surf. (2022) 26:174–83. doi: 10.1016/j.jtos.2022.08.009, PMID: 36067980

[4] Fukushima A. Roles of T-cells in the development of allergic conjunctival diseases. Cornea. (2007) 26:S36–40. doi: 10.1097/ICO.0b013e31812f6970 17881914

[5] Fukushima A. Roles of cytokines in the development of severe allergic conjunctival diseases: analyses using animal models. Nippon Ganka Gakkai Zasshi. (2004) 108:682–9. doi: 10.2174/1573412052953355, PMID: 15584353

[6] Iwamoto H Nishino K Magone TM Whitcup SM Yoshida O Yoshida H. Experimental immune-mediated blepharoconjunctivitis in rats induced by immunization with ragweed pollen. Graefes Arch Clin Exp Ophthalmol. (2000) 238:346–51. doi: 10.1007/s004170050363, PMID: 10853935

[7] Yoshida O Yoshida H Iwamoto H Nishino K Fukushima A Ueno H. Genetic background determines the nature of immune responses and experimental immune-mediated blepharoconjunctivitis (EC). Curr Eye Res. (1999) 18:117–24. doi: 10.1076/ceyr.18.2.117.5383, PMID: 10223655

[8] Liu YC Lin MTY Ng AHC Wong TT Mehta JS. Nanotechnology for the treatment of allergic conjunctival diseases. Pharmaceuticals. (2020) 13:351. doi: 10.3390/ph13110351, PMID: 33138064 PMC7694068

[9] Jauhonen HM Laihia J Oksala O Viiri J Sironen R Alajuuma P. Topical cis-urocanic acid prevents ocular surface irritation in both IgE -independent and -mediated rat model. Graefes Arch Clin Exp Ophthalmol. (2017) 255:2357–62. doi: 10.1007/s00417-017-3781-z, PMID: 28840310

[10] Chauhan SK Dana R. Role of Th17 cells in the immunopathogenesis of dry eye disease. Mucosal Immunol. (2009) 2:375–6. doi: 10.1038/mi.2009.21, PMID: 19532120 PMC2719854

[11] Liu YC Ng XW Teo EPW Ang HP Lwin NC Chan NSW. A biodegradable, sustained-released, tacrolimus microfilm drug delivery system for the Management of Allergic Conjunctivitis in a mouse model. Invest Ophthalmol Vis Sci. (2018) 59:675–84. doi: 10.1167/iovs.17-23066 29392313

[12] Fukushima A Yoshida H Iwamoto H Yoshida O Ueno H. The role of cellular immunity both in the induction and effector phases of experimental allergic blepharoconjunctivitis (EAC) in rats. Exp Eye Res. (1997) 65:631–7. doi: 10.1006/exer.1997.0362, PMID: 9367642

[13] Llewellyn BD. An improved Sirius red method for amyloid. J Med Lab Technol. (1970) 27:308–9. PMID: 4097661

[14] Barnett-Vanes A Sharrock A Birrell MA Rankin S. A single 9-colour flow cytometric method to characterise major leukocyte populations in the rat: validation in a model of LPS-induced pulmonary inflammation. PLoS One. (2016) 11:e0142520. doi: 10.1371/journal.pone.0142520, PMID: 26764486 PMC4713146

[15] Tong L Htoon HM Hou A Acharya RU Tan JH Wei QP. Acupuncture and herbal formulation compared with artificial tears alone: evaluation of dry eye symptoms and associated tests in randomised clinical trial. BMJ Open Ophthalmol. (2018) 3:e000150. doi: 10.1136/bmjophth-2018-000150, PMID: 30123846 PMC6093252

[16] Fukushima A Ozaki A Fukata K Ueno H. Differential expression and signaling of IFN-gamma in the conjunctiva between Lewis and Brown Norway rats. Microbiol Immunol. (2003) 47:785–96. doi: 10.1111/j.1348-0421.2003.tb03436.x, PMID: 14605445

[17] Fukushima A Fukata K Ozaki A Takata M Kuroda N Enzan H. Exertion of the suppressive effects of IFN-gamma on experimental immune mediated blepharoconjunctivitis in Brown Norway rats during the induction phase but not the effector phase. Br J Ophthalmol. (2002) 86:1166–71. doi: 10.1136/bjo.86.10.1166, PMID: 12234900 PMC1771302

[18] FUKUSHIMA A OZAKI A ISHIDA W VANROOIJEN N FUKATA K UENO H. Suppression of macrophage infiltration into the conjunctiva by clodronate liposomes in experimental immune-mediated blepharoconjunctivitis. Cell Biol Int. (2005) 29:277–86. doi: 10.1016/j.cellbi.2004.12.011, PMID: 15893479

[19] Bonini S Ghinelli E. The early and late phase of the ocular allergic reaction. Acta Ophthalmol Scand Suppl. (2000) 78:41. doi: 10.1034/j.1600-0420.2000.078s230041.x 11057349

[20] Ozaki A Ishida W Fukata K Fukushima A Ueno H. Phenotypic changes and inflammatory cell distribution in the cornea during development of experimental immune-mediated blepharoconjunctivitis. Jpn J Ophthalmol. (2004) 48:333–9. doi: 10.1007/s10384-004-0080-0, PMID: 15295657

[21] Ozaki A Fukushima A Fukata K Ueno H. Mast-cell activation augments the late phase reaction in experimental immune-mediated blepharoconjunctivitis. Graefes Arch Clin Exp Ophthalmol. (2003) 241:394–402. doi: 10.1007/s00417-003-0641-9, PMID: 12682842

[22] Ozaki A Fukushima A Ishida W Zinchuk O Fukata K Hayashi Y. Analysis of ag-presenting cells in the conjunctiva during the development of experimental immune-mediated blepharoconjunctivitis. Curr Eye Res. (2004) 29:277–86. doi: 10.1080/02713680490516873, PMID: 15590473

[23] Dhaliwal K Scholefield E Ferenbach D Gibbons M Duffin R Dorward DA. Monocytes control second-phase neutrophil emigration in established lipopolysaccharide-induced murine lung injury. Am J Respir Crit Care Med. (2012) 186:514–24. doi: 10.1164/rccm.201112-2132OC, PMID: 22822022 PMC3480527

[24] Lin SL Castaño AP Nowlin BT Lupher ML Jr Duffield JS. Bone marrow Ly6Chigh monocytes are selectively recruited to injured kidney and differentiate into functionally distinct populations. J Immunol. (2009) 183:6733–43. doi: 10.4049/jimmunol.0901473, PMID: 19864592

[25] Blöcher S Wilker S Sucke J Pfeil U Dietrich H Weimer R. Acute rejection of experimental lung allografts: characterization of intravascular mononuclear leukocytes. Clin Immunol. (2007) 124:98–108. doi: 10.1016/j.clim.2007.04.005, PMID: 17513175

[26] Lambiase A Normando EM Vitiello L Micera A Sacchetti M Perrella E. Natural killer cells in vernal keratoconjunctivitis. Mol Vis. (2007) 13:1562–7. PMID: 17893656

[27] Gorska MM. Natural killer cells in asthma. Curr Opin Allergy Clin Immunol. (2017) 17:50–4. doi: 10.1097/ACI.0000000000000327, PMID: 27841766 PMC5495007

[28] Mathias CB Guernsey LA Zammit D Brammer C Wu CA Thrall RS. Pro-inflammatory role of natural killer cells in the development of allergic airway disease. Clin Exp Allergy. (2014) 44:589–601. doi: 10.1111/cea.12271, PMID: 24397722 PMC3985123

[29] Bonini S Trocme SD Barney NP Brash PC Bloch KJ Allansmith MR. Late-phase reaction and tear fluid cytology in the rat ocular anaphylaxis. Curr Eye Res. (1987) 6:659–65. doi: 10.3109/02713688709034828, PMID: 3109809

[30] Sumagin R Brazil JC Nava P Nishio H Alam A Luissint AC. Neutrophil interactions with epithelial-expressed ICAM-1 enhances intestinal mucosal wound healing. Mucosal Immunol. (2016) 9:1151–62. doi: 10.1038/mi.2015.135, PMID: 26732677 PMC4935657

[31] Reyes NJ Saban DR. T helper subsets in allergic eye disease. Curr Opin Allergy Clin Immunol. (2014) 14:477–84. doi: 10.1097/ACI.0000000000000088, PMID: 25111509 PMC4167029

[32] Leonardi A Fregona IA Plebani M Secchi AG Calder VL. Th1- and Th2-type cytokines in chronic ocular allergy. Graefes Arch Clin Exp Ophthalmol. (2006) 244:1240–5. doi: 10.1007/s00417-006-0285-7, PMID: 16538446

[33] Stern ME Siemasko KF Niederkorn JY. The Th1/Th2 paradigm in ocular allergy. Curr Opin Allergy Clin Immunol. (2005) 5:446–50. doi: 10.1097/01.all.0000182547.60595.64, PMID: 16131922

[34] Nakashima T Niwano Y. Fungus as an exacerbating factor of atopic dermatitis, and control of Fungi for the remission of the disease. Atopic Dermatitis Dis Etiol Clin Manage. (2012). doi: 10.5772/26415

