# Supplementary figures and images for: Conjunctival infiltrates and cytokines in an experimental immune-mediated blepharoconjunctivitis rat model
Source: Front Med (Lausanne). 2023 Jun 28;10:1200589. doi: 10.3389/fmed.2023.1200589 (PMC10338090; doi:10.3389/fmed.2023.1200589)

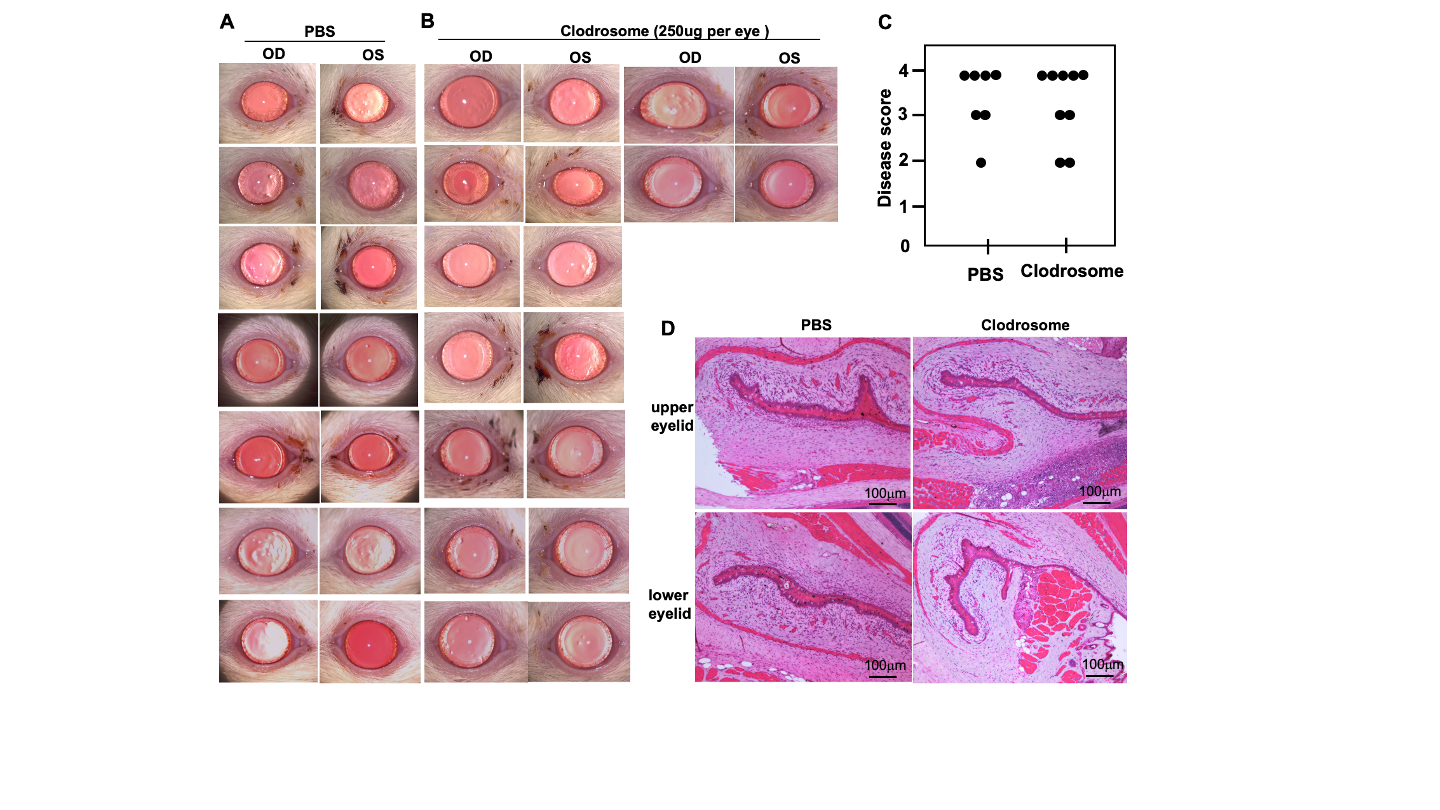

Supplement: SUPPLEMENTARY FIGURE 1 — (A,B) Slit lamp images of rat eyes. Rats were immunized with OVA in CFA. Two weeks later, 24hrs before OVA topical challenge, immunized rats were injected with either PBS or Clodrosome (250µg) at subconjunctiva. Both eyes of these rats were photographed with slit lamp. (C) The BC clinical disease score of these rats were graded as the following: each eye was assessed for eyelid excoriations and edema, and each findings was scored as 1 point. Scores from two eyes were added for each rat. (D) Representative H and E staining images of rat eyes from PBS and Clodrosome groups. [file Image_1.TIFF]
