# Supplementary material for: Conjunctival infiltrates and cytokines in an experimental immune-mediated blepharoconjunctivitis rat model
Source: Front Med (Lausanne). 2023 Jun 28;10:1200589. doi: 10.3389/fmed.2023.1200589 (PMC10338090; doi:10.3389/fmed.2023.1200589)
Supplement: Supplementary file 2 [file Data_Sheet_1.docx]

**Supplementary Table 1. Comparison of rat blepharoconjunctivitis models**

| **Study** | **Genetic background** | **Immunization reagents** | **Adjuvant** | **Immune cells identified and methods** | **Cytokine studied and methods** | **Type of immune response** |
| --- | --- | --- | --- | --- | --- | --- |
| Bonini S. et al 1987 ^1^ | SD | DNP-Ascaris | Alum | Neutrophils, eosinophils, lymphocyte, atypical epithelial cells in rat tears and acid-fast Giemsa stain | NA | NA |
| Yoshida H. et al, 1998 ^2^ | Lewis | OVA (adoptive transfer) | CFA | CD4/CD8 T cells in OVA primed Lymph nodes and Flow cytometry | Cytokine production in OVA primed lymph node cells: IFN-γ and ELISA and RT-PCR; IL-2, IL-4, IL-10 and RT-PCR | Th1 |
| Fukushima A. et al, 1998 ^3^ | Lewis | OVA, OVA 323-339 peptide and OVA 323-339-specific cell line (S816) adoptive transfer | CFA | S816 is CD4+ T cells and Flow cytometry | Cytokine production in OVA primed lymph node cells: IFN-γ and ELISA | Th1 |
| Yoshida O. et al 1998 ^4^ | Lewis and Fischer | OVA and OVA323-339 peptide | CFA | Same cellular profile of lymph node cells and Flow cytometry | IFN-γ in lymph node cells culture and NA | Th1 in both strains |
| Iwamoto H. et al, 1999 ^5^ | Lewis | OVA and OVA-primed lymph node cells | CFA | Total infiltrated cells in conjunctiva, and H and E staining | NA | NA |
| Fukushima A. et al 1999 ^6^ | Lewis | OVA, OVA(mPEG)_11_ and OVA primed lymph node ells from OVA(mPEG)_11_ treated rats | CFA | Total infiltrated cells in conjunctiva, and H and E staining | NA | NA |
| Yoshida A. et al 1999 ^7^ | Lewis and BN | OVA | CFA and ALUM | Total infiltration cells, and H and E | IFN-γ and IL-4 in lymph nodes cells and ELISA | Lewis: more Th1; BN: more Th2 |
| Iwamoto H. et al 2000 ^8^ | Lewis and BN | Ragweed pollen | CFA and ALUMN | Total infiltration cells and May-Giemsa | IFN-γ in lymph node cell culture and ELISA | Lewis: more Th1; BN: more Th2 |
| Fukushima A, et al 2003 ^9^ | BN | OVA-specific T cells | NA | T cells, macrophage and IHC; eosinophils and MBP | IL-2, 4, 5, 6, 10, 12p4, 13, IFN-γ, TGF-β, TNF-α, MCP-1, RANTES, MIP-1α, CCR3, CCR5, IP-10, CXCR3, and RT-PCR | Th1 and Th2 |
| Fukushima A, et al 2003^10^ | Lewis and BN | OVA-specific T cells | NA | Lymphocyte and eosinophils & H and E staining | IL-2, 4, 5, 10, IFN-γ and RT-PCR | Lewis: more Th1; BN: more Th2 |
| Ozaki A. et al 2003 ^11^ | BN | OVA-specific T cells | NA | Eosinophils, mononuclear cells and neutrophils & H and E, MG and PAS staining | RANTES and Eotaxin and RT-PCR | NA |
| Ozaki A, et al 2004 ^12^ | BN | OVA-specific T cells | NA | Macrophage & IHC; mononuclear cells and eosinophils & MG staining | NA | NA |
| Ozaki A, et al 2004 ^13^ | BN | OVA | CFA | Macrophage, T cells, dendritic cells in cornea & IHC | NA | NA |
| Current study | SD | OVA | CFA | T cells, mono-macrophage, neutrophils, NK cells & Flow cytometry; Eosinophils & Siris Red staining | IL-2, IL-4, IL-6, IL-17A, IFN-γ, and TNF-α and Multiplex assay | More Th1 response |

SD: Sprague Dawley; BN: Brown Norway; OVA: Ovalbumin; H and E: Hematoxylin and eosin; MG and PAS: May-Giemsa and Periodic Acid-Schif

References

1. Bonini S, Trocmé SD, Barney NP, Brash PC, Bloch KJ, Allansmith MR. Late-phase reaction and tear fluid cytology in the rat ocular anaphylaxis. *Curr Eye Res*. May 1987;6(5):659-65. doi:10.3109/02713688709034828

2. Yoshida H, Yoshida O, Iwamoto H, et al. Analysis of effects of stimulation in vitro of ovalbumin primed lymph node cells on adoptive transfer of experimental immune mediated blepharoconjunctivitis in Lewis rats. *Br J Ophthalmol*. Oct 1998;82(10):1189-94. doi:10.1136/bjo.82.10.1189

3. Fukushima A, Nishino K, Yoshida O, Ueno H. Characterization of the immunopathogenic responses to ovalbumin peptide 323-339 in experimental immune-mediated blepharoconjunctivitis in Lewis rats. *Curr Eye Res*. Aug 1998;17(8):763-9.

4. Yoshida O, Yoshida H, Iwamoto H, Nishino K, Fukushima A, Ueno H. Comparison of genetic susceptibility to experimental allergic/immune-mediated blepharoconjunctivitis between Lewis and Fischer rats. *Graefes Arch Clin Exp Ophthalmol*. Nov 1998;236(11):859-64. doi:10.1007/s004170050171

5. Iwamoto H, Yoshida H, Yoshida O, Fukushima A, Ueno H. Inhibitory effects of FK506 on the development of experimental allergic/immune-mediated blepharoconjunctivitis in Lewis rats by systemic but not by topical administration. *Graefes Arch Clin Exp Ophthalmol*. May 1999;237(5):407-14. doi:10.1007/s004170050252

6. Fukushima A, Nishino K, Yoshida H, Takata M, Ueno H. Suppression of induction of experimental immune mediated blepharoconjunctivitis by tolerogenic conjugates of the antigen and monomethoxypolyethylene glycol. *Br J Ophthalmol*. Aug 1999;83(8):973-9. doi:10.1136/bjo.83.8.973

7. Yoshida O, Yoshida H, Iwamoto H, Nishino K, Fukushima A, Ueno H. Genetic background determines the nature of immune responses and experimental immune-mediated blepharoconjunctivitis (EC). *Curr Eye Res*. Feb 1999;18(2):117-24. doi:10.1076/ceyr.18.2.117.5383

8. Iwamoto H, Nishino K, Magone TM, et al. Experimental immune-mediated blepharoconjunctivitis in rats induced by immunization with ragweed pollen. *Graefes Arch Clin Exp Ophthalmol*. Apr 2000;238(4):346-51. doi:10.1007/s004170050363

9. Fukushima A, Ozaki A, Fukata K, Ishida W, Ueno H. Ag-specific recognition, activation, and effector function of T cells in the conjunctiva with experimental immune-mediated blepharoconjunctivitis. *Invest Ophthalmol Vis Sci*. Oct 2003;44(10):4366-74. doi:10.1167/iovs.02-1323

10. Fukushima A, Ozaki A, Fukata K, Ueno H. Differential expression and signaling of IFN-gamma in the conjunctiva between Lewis and Brown Norway rats. *Microbiol Immunol*. 2003;47(10):785-96. doi:10.1111/j.1348-0421.2003.tb03436.x

11. Ozaki A, Fukushima A, Fukata K, Ueno H. Mast-cell activation augments the late phase reaction in experimental immune-mediated blepharoconjunctivitis. *Graefes Arch Clin Exp Ophthalmol*. May 2003;241(5):394-402. doi:10.1007/s00417-003-0641-9

12. Ozaki A, Fukushima A, Ishida W, et al. Analysis of Ag-presenting cells in the conjunctiva during the development of experimental immune-mediated blepharoconjunctivitis. *Curr Eye Res*. 2004 Oct-Nov 2004;29(4-5):277-86. doi:10.1080/02713680490516873

13. Ozaki A, Ishida W, Fukata K, Fukushima A, Ueno H. Phenotypic changes and inflammatory cell distribution in the cornea during development of experimental immune-mediated blepharoconjunctivitis. *Jpn J Ophthalmol*. 2004 Jul-Aug 2004;48(4):333-9. doi:10.1007/s10384-004-0080-0
